# A novel traveling-wave-based detection and self-healing approach for series faults in smart grids under renewable energy uncertainty

**DOI:** 10.1038/s41598-025-27858-1

**Published:** 2025-12-05

**Authors:** Mahmoud A. Elsadd, Amina I. Elezzawy, Tamer A. Kawady, Nagy I. Elkalashy, Mahmoud M. Elgamasy

**Affiliations:** 1 Electrical Engineering Department, Faculty of Engineering, Damanhour University, Damanhour, Egypt; 2 Electrical Engineering Department, Faculty of Engineering, Menoufia University, Menoufia, Egypt

**Keywords:** Fault detection, Fault management, Open conductor, Renewable generation uncertainty, Series fault, Traveling wave, Energy science and technology, Engineering

## Abstract

Conventional protective devices cannot reliably detect series faults at the far end of the distribution feeder, such as open-conductor faults, downed conductor faults from the load side, and downed conductor faults from the source side when associated with high-impedance conditions. This limitation is especially challenging in the presence of distributed generation units. This paper introduces an innovative scheme for detecting series faults using traveling wave voltage measurements. This scheme identifies faults by comparing the polarities of the first-arriving voltage waves at the lateral ends. During such faults, the polarity at the lateral end downstream of the fault differs from the others. Building on this principle, a fault management scheme is proposed that combines polarity differences with a two-terminal traveling-wave scheme to both locate the fault and restore service. The scheme is validated on the IEEE 33-bus system using PSCAD simulations. The results confirm that the proposed scheme is sensitive to all types of series faults, whether or not distributed generation (DG) units are connected, and remains secure under normal operating conditions such as load switching and capacitor bank connection. Furthermore, the scheme accommodates the uncertainty inherent in renewable energy sources, making it effective across the full range of power contributions—from zero (when disconnected from the grid) to full output. The results confirm that proposed scheme provides high reliability in detecting the open-conductor faults and identifying faulted laterals regardless of synchronization misalignment between measurement points. It accurately determines the faulted section for synchronization deviations up to 3 µs, while deviations exceeding 1 µs may affect accuracy under busbar fault conditions. A verification step using fault indicators at secondary substations ensures correct section isolation even under significant synchronization deviations. Overall, the proposed algorithm outperforms existing methods, particularly in networks with DG units.

## Introduction

### Motivation

 The continuous increase in electricity consumption has created an urgent need to expand generation capacity. However, this expansion often brings about higher production costs, environmental impacts, and accelerates global warming. To address the escalating expenses linked to energy transmission and distribution losses, planners have increasingly turned to DG systems as viable solutions within power distribution networks. Extensive research has investigated the optimal integration and utilization of DG technologies^1,2^. Based on findings from the Electric Power Research Institute and Natural Gas Institutions, DG units are expected to contribute around 30% of the total electricity generation in the near future^3,4^. Among the most widespread DG technologies are wind and solar power systems, which are considered promising in modern grid planning due to their sustainability and cost-effectiveness. Nevertheless, the incorporation of DG units introduces new challenges for the protection infrastructure of active distribution systems^5^. Their presence can undermine the reliability of protection schemes, leading either to maloperations (insecurity) or failures in detecting critical faults (lack of dependability)^6^.

Traditional protective devices have difficulty detecting open conductors at the remote end of distribution feeders, as well as downed conductor faults originating from either the load side or the source side, particularly when high fault resistance is involved. Furthermore, modern protection functions also exhibit limitations, especially in detecting series faults in the presence of DG units, as discussed in the next subsection.

In recent years, distribution networks have been rapidly evolving into smart distribution systems. These systems are equipped with fast fault detection and self-healing capabilities, enabling them to isolate the faulted zone and restore power to unaffected areas through alternative paths using tie switches—without human intervention^7^. Timely isolation of the faulted zone is essential to initiate repairs and restore service to all customers. Motivated by this, the present paper addresses a specific gap: the development of both an effective fault detection function and a management control method for handling series faults in distribution systems with DG units. This remains a key challenge for protection and control engineers.

### Literature survey

The detection of series faults, including downed conductors associated with high-impedance faults in distribution systems, has been investigated in several studies, as summarized below. In^8^, a method that utilizes voltage measurements at medium voltage/low voltage stations to locate downed conductor faults is presented. It follows a two-step approach: first, determining the sensor’s position relative to the fault, and second, identifying the faulted section based on the network topology. However, this method is specifically designed for radial distribution systems. In^9^, a high-impedance earth fault locator function for distribution systems are presented. In^10^, a method for detecting earth faults in distribution systems is presented, employing a fuzzy measure fusion criterion that quantifies the similarity between real-time and historical fault data using fuzzy c-means clustering and a hierarchical evaluation index system to locate the faulty feeder. However, these methods of^8–10^ are designed for high-impedance faults that do not involve broken conductors. In^11^, a hybrid method is introduced that integrates discrete wavelet transform with a neural network for distinguishing downed conductor faults from normal switching operations. However, it requires a high sampling frequency and extensive training data, and its performance may lead to false classifications in real-world fault scenarios. In^12^, distributed low-voltage transformer measurements with global system for mobile communications are used to detect open-conductor faults. In^13^, a method for detecting open-conductor faults based on time shifts in three-phase line currents is presented, in conjunction with traditional overcurrent protection, effectively covering the entire length of radial systems. However, the method in^13^ is tailored specifically for radial distribution systems.

In^14,15^, the ratio of negative-sequence to positive-sequence current components is used to detect open-conductor faults. However, the change in this ratio becomes insignificant when the fault occurs far from the primary substation. Additionally, accurate threshold settings are required to distinguish between open-conductor faults and normal unbalanced conditions. In^16^, distributed measurement devices are installed at the beginning of each section in the system to measure phase and zero-sequence currents. Fault detection relies on identifying a drop in phase current to zero, while faulted section identification is based on analyzing current characteristics during the fault. However, the method in^16^ requires a large number of devices at the medium-voltage (MV) side, equal to the number of system sections. In^17,18^, smart meters are installed on the low-voltage side of distribution transformers, offering a cost-effective solution by avoiding the need for measurement devices on the high-voltage side. In^19^, a series fault detection system based on voltage unbalance along distribution feeders is proposed, utilizing voltage sensors at all feeder branches. The system uses the V2/V1 ratio (50–100%) to detect broken conductors and the V0/V1 ratio (> 100%) to identify downed conductors in contact with the ground. However, the approaches in^17–19^ are not secure and may malfunction if the fault occurs on the low-voltage side downstream of the measurement point. To address this issue^20^, presents a new voltage unbalance ratio with an innovative threshold value based on a fault analysis theorem, integrated with a blocking function that monitors the phase current and a proposed coordination time interval longer than the total fuse clearing time). This approach effectively distinguishes internal series faults from external shunt/series faults and abnormal conditions. This approach also offers a cost-effective solution by using only the magnitudes of measured voltages, eliminating the need for pre-fault data, phase angles, or topological information, while enabling a robust threshold and an autonomous control. In addition, the performance limitations of protective devices installed in distribution systems were recorded and analyzed under various series fault scenarios in^20^ by the same authors of this paper. However, none of the techniques^12–20^ discussed above consider the impact of integrating DG units into the system under open-conductor faults.

### Key challenges, contributions, and innovations

Detecting and locating series faults remains a significant challenge in the field. While shunt faults are relatively easy to detect due to the high current levels observed at the primary substation, series faults are more difficult to identify because they cause only small variations in current. These variations may not be detected by substation relays. Furthermore, if the conductor falls at the far end of the distribution feeder, the resulting negative-sequence current component may be too small for reliable detection. Detecting these faults is crucial due to their potentially harmful impacts on both humans and equipment. For instance, a series fault—such as an open conductor—can generate a negative-sequence current component that produces higher temperatures than the corresponding positive-sequence component, potentially damaging dynamic equipment such as induction motors. Additionally, if the conductor falls from either the source or load side, it can pose serious safety hazards, including electric shocks or fire risks. Therefore, implementing a self-healing scheme within the distribution system is essential to address these issues effectively. However, existing self-healing approaches are primarily designed for radial distribution systems. The following points highlight the challenges faced by these approaches due to the integration of DG units into power distribution systems, particularly when a series fault occurs upstream of a highly penetrated DG unit.

- The current at agents downstream of a series fault—such as an open-conductor fault or an open-conductor fault with a fallen conductor from the source side—will not drop to zero, as it typically does in conventional radial distribution system configurations. In such cases, existing methods based on phase quantities face challenges in the presence of DG unit.
- The ratio of the negative-sequence current to the positive-sequence current (*I₂*/*I₁*) decreases during series faults—such as an open-conductor fault or an open-conductor fault with a fallen conductor from the source side—because of an increase in positive-sequence current caused by the presence of DG unit. In such cases, existing methods that rely on the *I₂*/*I₁* ratio encounter challenges in the presence of DG unit.
- The return current from a corner of the delta-connected primary winding of a low-voltage transformer (with a delta/star-earthed configuration) downstream of an open-conductor fault (without a fallen conductor) will not be zero due to the presence of DG unit behind the fault. As a result, the voltage unbalance ratios measured at agents downstream of the fault are reduced. Consequently, existing methods that rely on voltage unbalance and predefined threshold values face difficulties in detecting open-conductor faults (without a fallen conductor) in the presence of DG unit.

Therefore, conventional series fault detection and self-healing approaches encounter operational challenges in the presence of DG units. Accordingly, series fault detection and self-healing under DG units-integrated conditions are identified as critical challenges and constitute the key contributions of this work. To address these challenges, an innovative scheme is proposed for detecting critical series faults using traveling wave voltage measurements, taking into account the uncertainty associated with DG units. The scheme detects faults by comparing the polarities of the first-arriving voltage waves at the lateral ends (agents), where polarity differences at the downstream agent indicate the presence of a fault. A management scheme based on these polarities, combined with the two-terminal traveling wave method, is then used to identify the faulted lateral, faulted section, locate the fault, and restore service. The main advantages of the proposed scheme are as follows:

- It requires only voltage transducers at the lateral ends.
- It does not rely on predefined threshold values, as it detects series faults through voltage polarity comparison.
- It maintains security under critical conditions such as load switching and capacitor bank operations.
- It is specifically designed for active distribution networks that incorporate DG units, ensuring dependability during series faults in such systems.
- It effectively handles the uncertainties associated with renewable DG units without the need for adaptive procedures.

Validation using PSCAD simulations on the IEEE 33-bus system confirms the proposed scheme’s high sensitivity to various series faults—both in the presence and absence of distributed generation—while maintaining security under normal operating conditions such as load switching and capacitor bank operations. Furthermore, the results demonstrate superior performance compared to existing methods, particularly in systems with distributed generation.

The rest of the paper is organized as follows: Section II discusses voltage traveling waves caused by open-conductor faults. Section III introduces the proposed scheme for series fault detection and management. Validation results of the proposed scheme are presented in Section IV. Section V provides a security assessment of the proposed series fault detection method. Finally, Section VI presents the conclusion of the paper.

## Voltage traveling waves caused by open-conductor faults

**Fig. 1:**
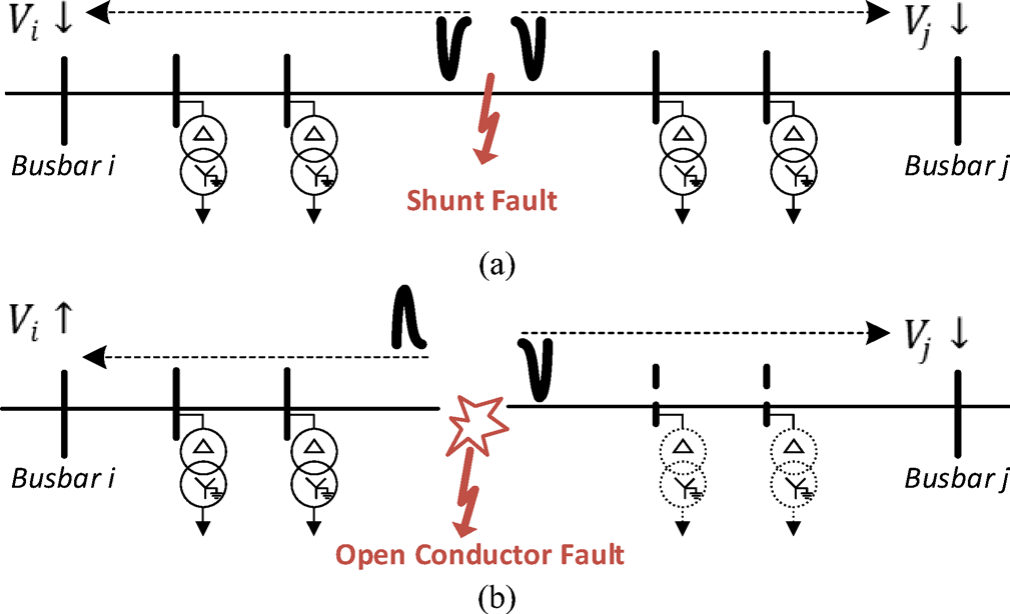
Generated traveling waves due to (a) shunt faults and (b) series fault (open-conductor) fault. a.

Traveling waves produced by faults have long been used to locate faults, particularly for shunt faults. However, no research has focused on using wave polarity to detect series faults. In a shunt fault, the voltage of the faulted phase drops to low levels along the feeder on both the upstream and downstream sides of the fault point. The resulting traveling voltage waves will have the same polarity on both sides, as shown in Fig. 1a. In contrast, in series faults such as open-conductor faults, the voltage of the faulted phase increases upstream and decreases downstream due to the disconnected loads caused by the open conductor. In this case, the traveling waves at both ends exhibit opposite polarities, as illustrated in Fig. 1b. These criteria can be used to detect open-conductor faults in the distribution system.

This paper introduces a proposed scheme for detecting series faults using traveling wave voltage measurements. In this scheme, the polarities of the measured waves at both the main substation and lateral ends are compared to detect faults. A fault management scheme is then proposed, relying on these polarities and the two-terminal traveling wave method to determine the faulted section and locate the fault precisely.

## Proposed scheme for series faults detection and management

**Fig. 2:**
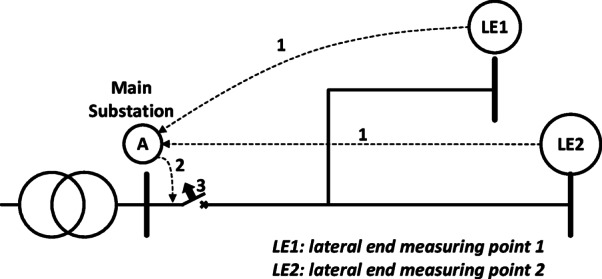
Communication between the agents at lateral ends and the primary substation.

The proposed scheme is designed to detect and manage open-conductor faults, addressing the limitations of existing methods under DG integration. Open-conductor fault detection, faulted lateral identification, and faulted section determination are achieved by deploying agents to monitor voltages at the primary substation (PS) and at the lateral ends, as illustrated in Fig. 2. All agents are strategically connected to the primary substation at the feeder outlet via communication channels to transmit their status information. Faulted section isolation is accomplished through communication between the primary substation and the secondary substations located upstream and downstream of the fault point. Each agent, regardless of its level, is represented as an intelligent entity capable of communicating with other agents in preceding and succeeding levels^21,22^. Additional details regarding the agent implementation are provided in^23^ by the same authors as this paper.

### The proposed series faults detection

**Fig. 3:**
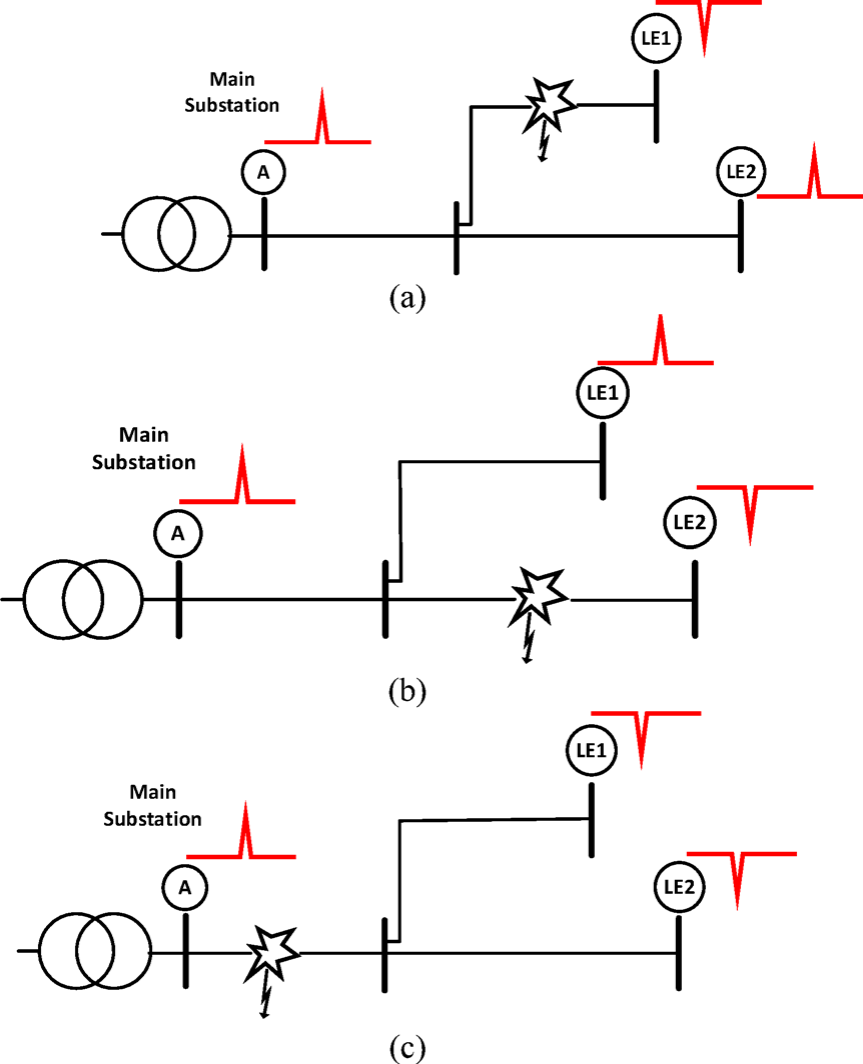
Examples of open-conductor faults at different sections: (**a**) Lateral 1, (**b**) Lateral 2, and (**c**) the section upstream of these two laterals.

This scheme differentiates between normal system operation (voltage drop and swell cases) and various series faults, including open-conductor faults, open-conductor faults with a fallen conductor from the source side, and open-conductor faults with a fallen conductor from the load side. This differentiation is achieved by monitoring the polarities of the first-arrival voltage waves detected by agents located at designated points, including the primary substation. The method for detecting this type of fault relies on identifying polarity discrepancies between downstream and upstream agents. If at least one received polarity opposes another, confirming the presence of a series-fault condition, the main feeder is promptly isolated. Various fault scenarios are illustrated in Fig. 3, encompassing faults occurring at the first lateral (Fig. 3a), the second lateral (Fig. 3b), and the section upstream of these lateral points (Fig. 3c).

The polarities of the first-arrival voltage waves at the agents are obtained by applying a signal-derivative scheme^24^ (as a feature extraction technique) on both aerial mode 1 and zero mode to verify the redundancy principle. The use of the zero mode is more secure during the fault detection stage, since no zero-sequence component exists prior to the fault occurrence. However, the waves extracted from aerial mode 1 are more accurate during the faulted section estimation (fault location determination) stage, as they exhibit sharper waveforms. Therefore, the voltage signals are decomposed using Clarke modal transformation^24^, as:

$$\:{v}_{\alpha\:}=\frac{2}{3}\left({v}_{a}-\frac{1}{2}{v}_{b}-\frac{1}{2}{v}_{c}\right)$$

$$\:{v}_{\beta\:}=\frac{2}{3}\left(0+\frac{\sqrt{3}}{2}{v}_{b}-\frac{\sqrt{3}}{2}{v}_{c}\right)$$

1 $$\:{v}_{0}=\frac{2}{3}\left({\frac{1}{2}v}_{a}+\frac{1}{2}{v}_{b}+\frac{1}{2}{v}_{c}\right)$$

where $\:{v}_{\alpha\:}$, $\:{v}_{\beta\:}$, and $\:{v}_{0}$ are defined as aerial mode 1, aerial mode 2, and zero mode voltages, respectively. The validation of the proposed criterion is explained as follows:

#### The introduced criterion validation in radial distribution systems

**Fig. 4:**
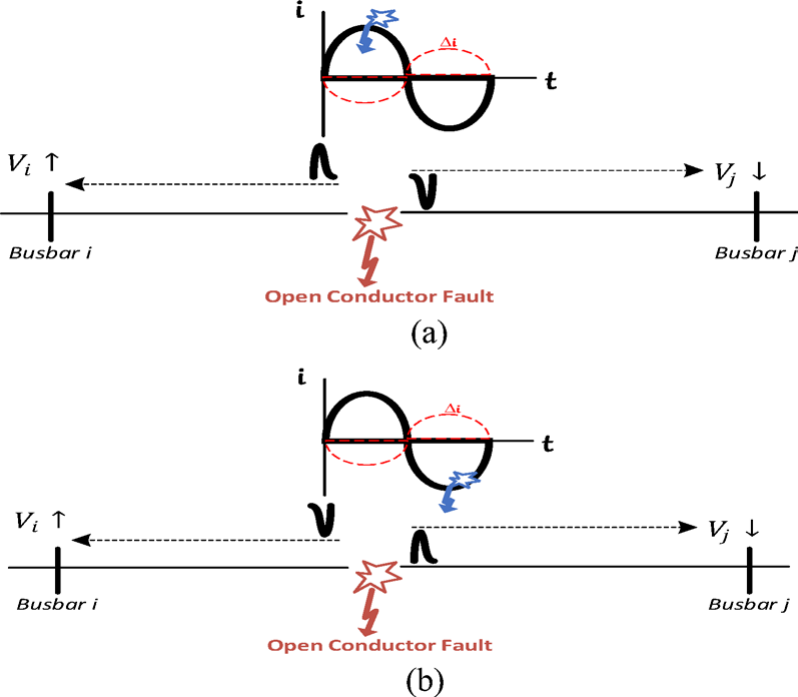
Generated traveling waves during an open-conductor fault in the distribution system without DG, considering different fault instants: (**a**) at the positive half-cycle and (**b**) at the negative half-cycle of the current waveform at the fault point.

The validation of the proposed criterion, based on the polarities of the first-arrival voltage waves measured at the installed agents in radial distribution systems without DG units, is presented as follows:

The physical meaning of these different polarities is that the voltage at the agent point upstream of the open-conductor fault increases, while it decreases at agents downstream of the same fault. This is because the voltage change (∆v) at upstream agents is inversely proportional to the current change (∆i), whereas ∆v at downstream agents is directly proportional to ∆i, as illustrated in Table 1. The polarities of the first-arrival wave at agents upstream and downstream of the fault could be positive or negative, but they are always opposite to each other. The positive or negative polarity (upward or downward change direction) depends on the fault instant with respect to the current waveform at the fault point before the fault occurrence, as illustrated in Fig. 4. In other words, if the fault occurs when the current at the fault point (prior to the fault occurrence) was in the positive half-cycle, then the current decreases, making ∆i negative at both upstream and downstream agents. Consequently, the voltage change (∆v) at upstream agents is positive, while the voltage change at downstream agents is negative, as shown in Table 1. Similarly, the polarities of the current and voltage are indicated in Table 1 if the fault instant occurs during the negative half-cycle of the current waveform.

For example, if an open-conductor fault occurs when the current at the fault point is in the positive half cycle, then the faulted phase currents at all agents decrease by |∆i|, as shown in the first row of Table 1. Consequently, the faulted phase voltages at agents upstream of the fault point increase by |∆v|. Therefore, one can conclude that the voltage change (∆v) at agents upstream of the fault (such as Agent *i*) is directly proportional to -∆i, as indicated in the third row of Table 1. However, the faulted phase voltages at agents downstream of the fault point decrease by |∆v| due to the disconnection of these agents from the grid (source side). Therefore, ∆v at downstream agents (such as Agent *j*) is directly proportional to ∆i, as illustrated in the third row of Table 1. As a result, the polarity of the first-arrival wave at Agent *i* is positive, whereas the first-arrival wave’s polarity at Agent *j* is negative, as shown in the third row of Table 1. The opposite occurs if an open-conductor fault takes place during the negative half-cycle, as illustrated in the fourth row of Table 1.

**Table 1:** Polarity changes in current and voltage at upstream and downstream agents for various fault instants in radial distribution systems.

Quantity		Bus i	Bus j
∆i	Positive cycle	**-**	**-**
	Negative cycle	**+**	**+**
∆v	Positive cycle	**+**	**-**
	Negative cycle	**-**	**+**

#### The introduced criterion validation in distribution systems with DG presence

The validation of the proposed criterion, which is based on the polarities of the first-arrival voltage waves at agents in distribution systems with DG units, is explained as follows:

First, if the open-conductor fault is downstream of the DG location, the validation of the presented criterion is similar to that in the previous subsection with radial distribution systems. Second, if the open-conductor fault occurs upstream of the DG location, the validation of the presented criterion is based on the direction of the pre-fault current (*I*_*pre*_) in the faulted section, as shown in Table 2. Furthermore, in this case, the polarities of the first-arrival wave at agents located upstream and downstream of the fault point may be positive or negative, but they are always opposite to each other. This is because the voltage change at upstream agents (∆v_i_) is inversely proportional to the current change upstream of the fault (∆i_busi_). In contrast, the voltage change at downstream agents (∆v_j_) is inversely proportional to the current change at the DG (∆i_DG_), which is inversely proportional to ∆i_busi_, as illustrated in Table 2. Therefore, the ∆v_j_ is directly proportional to the ∆i_busi_.

**Table 2:** Polarity changes in current and voltage at upstream and downstream agents for various fault instants in distribution systems with DG downstream of the fault.

Direction of I_pre_	Quantity		Bus i	Bus j
Downstream	∆i_busi_	+ve cycle	-	
		-ve cycle	+	
	∆V	+ve cycle	+	-
		-ve cycle	-	+
Upstream	∆i_busi_	+ve cycle	+	
		-ve cycle	-	
	∆V	+ve cycle	-	+
		-ve cycle	+	-

note: ΔV_i_ α -Δi_busi_, ΔV_j_ α -Δi_DG_ where Δi_DG_ α -Δi_busi_.

### The proposed series faults management

After carrying out the fault detection stage, the next step is the fault management process. To ensure reliable fault management, the smallest faulted area must be isolated to restore service continuity. The challenge lies in identifying the faulted lateral and subsequently determining the faulted section within it. The proposed methods are outlined in the following subsections.

#### The proposed faulted lateral determination

During the fault detection stage, the faulted lateral can be identified based on the observed polarities. As described previously, the branch containing the fault exhibits opposite polarities between the backward and forward traveling waves. For example, in the simple system shown in Fig. 2, there are three possible fault locations. If a fault occurs in Lateral 1, as shown in Fig. 3a, the wave extracted at lateral end 1 (LE1) is opposite in polarity to those from all other measurement points, indicating that the fault lies in this branch. Similarly, Fig. 3b illustrates a fault in Lateral 2. Conversely, if the measurement points of both laterals produce waves that are opposite in polarity to the wave extracted from the primary substation at point A, it indicates that the fault is located in the path between point A and the intersection point, as shown in Fig. 3c. The complete sequence of the proposed method for identifying the faulted lateral using the modified control is presented in Subsubsection C.

#### The faulted section determination

**Fig. 5:**
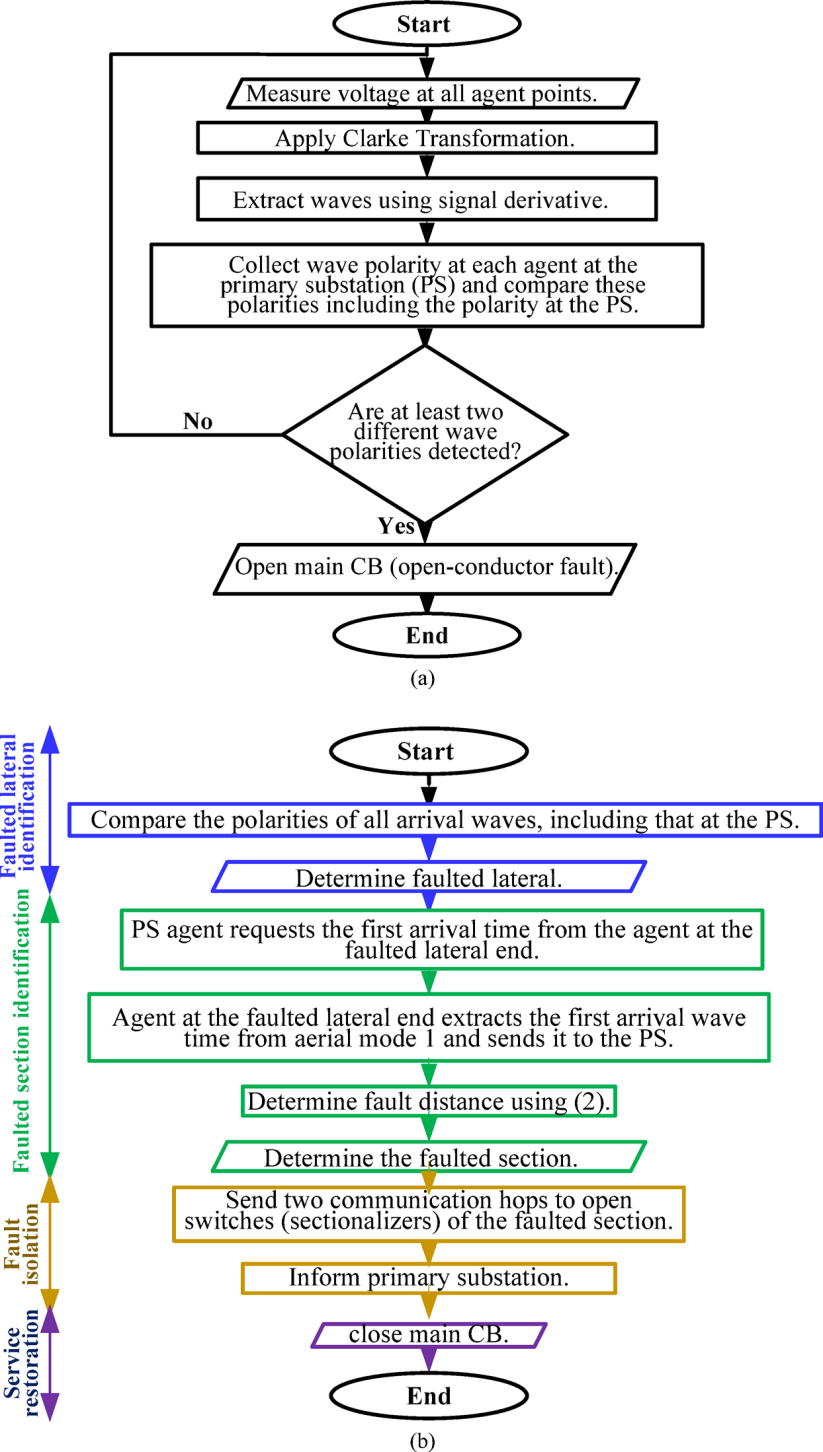
The proposed scheme for series fault: (**a**) detection and (**b**) management.

After identifying the faulted lateral in the distribution system, it is necessary to determine the faulted section. The conventional two-terminal traveling wave equation for fault location estimation is employed between the agent at the faulted lateral end and that at the primary substation, using the extracted waves from aerial mode 1, which produces sharp waves, as follows:

2 $$\:d=\frac{{L}_{Aj}\:-\:\nu\:\left({t}_{A}\:-{\:t}_{j}\right)}{2}$$

where *d* is the fault distance, calculated from the point at the end of the faulted lateral; *t*_*A*_ and *t*_*j*_ are the times of the captured first-arrival waves at the primary substation agent *A* and at the lateral end *j*, respectively; and *v* is the traveling-wave velocity. The calculated distance helps determine which section is faulted along the path connecting busbars *A* and *j*. The primary substation agent sends a signal (hop) to the faulted lateral-end agent, requesting the time of the first-arrival wave. This agent responds with its arrival time, which is used for calculating the distance to the fault and determining the faulted section. The primary substation then sends hops to the selected section’s forward and backward switches for isolation. If the fault is located close to one of the busbars, the calculated distance may not accurately identify the faulted section. Thus, if the calculated distance (*d*) lies between 10% and 90% of the section length, the faulted section is confirmed and isolated. If the calculated distance lies between 0% and 10% of the section length, it should be isolated along with its upstream neighbouring section. If the calculated distance lies between 90% and 100% of the section’s length, it should be isolated along with its downstream neighbouring section. In the fault case shown in Fig. 3c, where more than one lateral agent has an opposite wave compared to that at the primary substation, a predefined lateral-end agent must be selected to calculate the distance using its arrival time. The proposed method is summarized in the flow chart in Fig. 5. Series fault detection is illustrated in Fig. 5a, whereas series fault management—including the steps of faulted lateral identification, faulted section identification, faulted section isolation, and service restoration—is illustrated in Fig. 5b.

### The proposed centralized control strategy

**Fig. 6:**
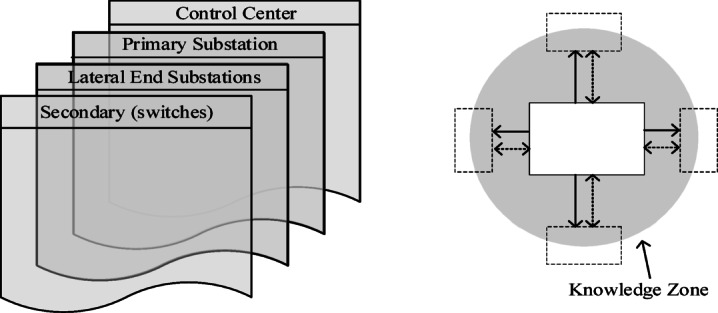
Information hierarchy layers of the proposed scheme.

The proposed scheme relies on a modified centralized control strategy. The fault management information hierarchy consists of four levels, as shown in Fig. 6. These levels are the control center, the primary substation, the lateral-end substation, and the secondary substation. On the other hand, the presented fault detection methodology is based only on the information from the first three levels, without the need for the secondary substation level. Each agent at any level is represented as an object capable of communicating with other agents in preceding and succeeding levels.

The proposed detection process begins when lateral-end substations capture significant surges. Their agents then send the wave polarities to the primary substation. This step requires *n* communication hops, where *n* is the number of lateral-end substations. A comparison between these polarities is made at the primary substation. If two different wave polarities are detected, the primary substation sends a hop to the control center. The control center then sends a hop to the primary substation to open the main circuit breaker. Therefore, the total number of communication hops needed for the detection stage is *n* + 2.

**Fig. 7:**
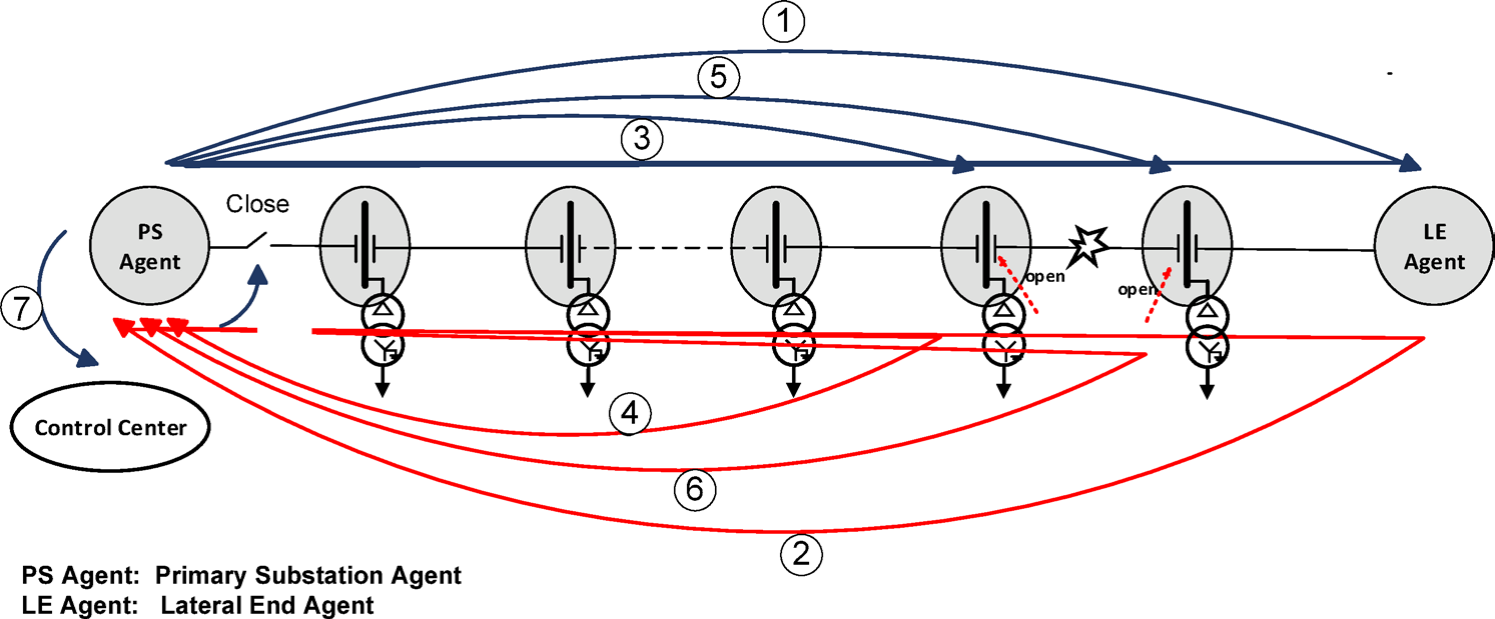
Communications between the agent at the PS and the agents at the faulted lateral end, as well as those at the secondary substations near the faulted section.

Next, after the circuit breaker opens, the proposed fault management process is initiated. A communication signal is sent from the agent at the primary substation to the agent at the lateral end having a different wave polarity to request the time of the first-arrival voltage wave. This marks the first communication hop in the fault management stage. The agent at this lateral end then sends its arrival time back to the primary substation agent to identify the faulted section. This adds an additional communication ho After identifying the faulted section, the primary substation agent communicates sequentially with the two agents at the secondary substations associated with that section to open their switches near the fault, thereby isolating the faulted area. These secondary substations sequentially send confirmations indicating that their switches near the fault have been opened. This process requires four additional communication hops. Finally, the primary substation restores the service and sends a signal (the last communication hop) to the control center to update it on the new system configuration. Therefore, the modified centralized control strategy requires seven communication hops in total, as illustrated in Fig. 7. The proposed fault management scheme demonstrates promising performance compared to existing techniques^22,23^, where the evaluation of management control strategies is based on calculating the total latency time according to the number of communication hops required to restore the system.

**Fig. 8:**
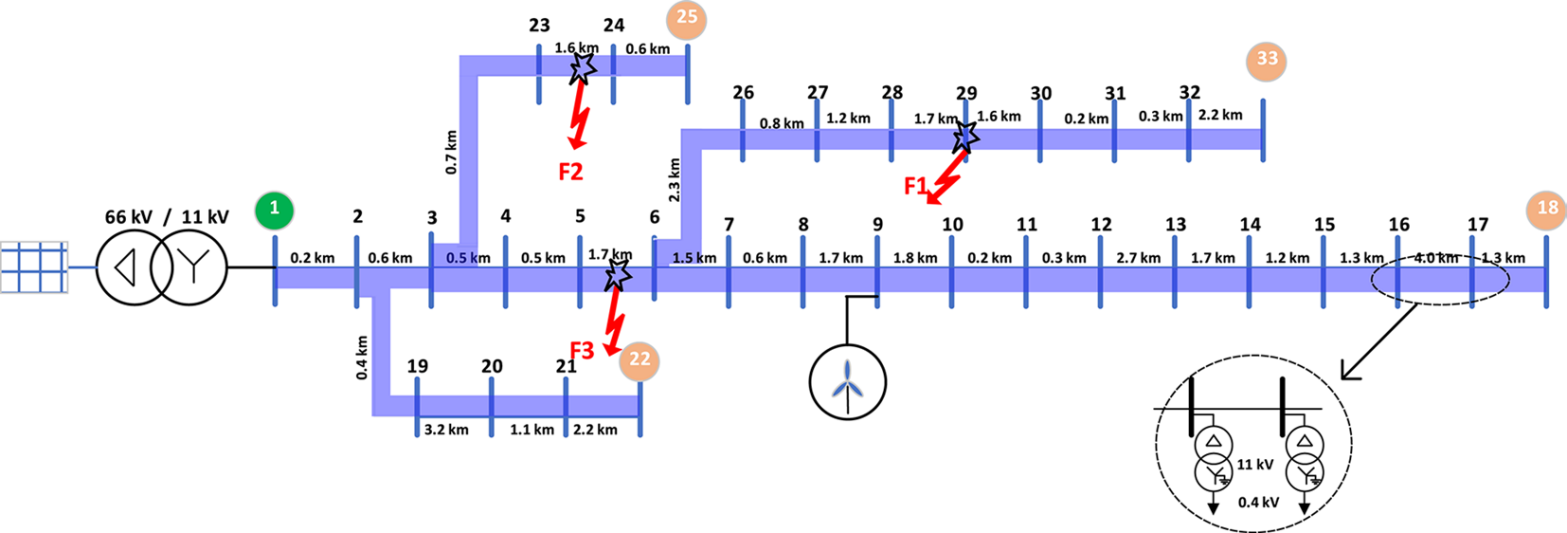
The IEEE 33-bus distribution system showing the locations of the primary substation agent at busbar 1 and the lateral end agents at busbars 18, 22, 25, and 33.

## Validation results of the proposed scheme’s dependability

The IEEE 33-bus, 11 kV overhead radial distribution feeder has been selected to validate the proposed scheme, as indicated in Fig. 8. For convenience, the sections lengths are presented as shown in Fig. 8, and more details are available in^22,23^. The proposed scheme aims to reliably detect series faults and determine the faulted section while using a limited number of measurement points. Voltage-measurement points are installed at busbar 1 (the primary substation) and at busbars 18, 22, 25, and 33 (the lateral ends). The selected test system is simulated using PSCAD software, and the feeder sections are represented by frequency-dependent models. The measured signals are sampled at a rate of 1 MHz.

Three different open-conductor fault locations are simulated to evaluate the proposed scheme, as depicted in Fig. 8. The first fault (F1) occurs at busbar 29, the second fault (F2) occurs in Section 23–24, 1.5 km from busbar 3, and the third fault (F3) occurs in Section 5–6, 1.9 km from busbar 3.

### The fault detection process

**Fig. 9:**
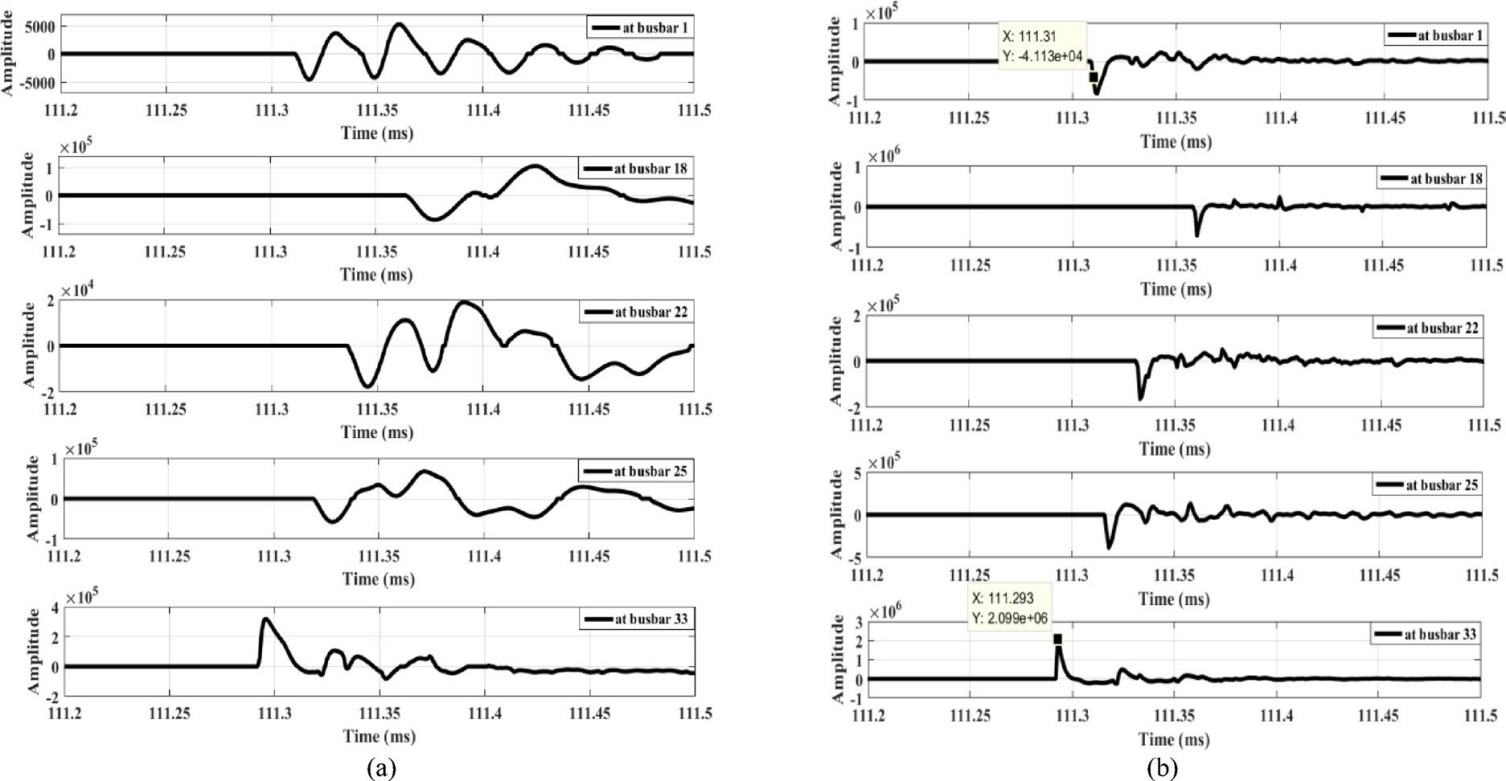
Extracted traveling waves under a fault F1: (**a**) Zero mode, (**b**) Aerial mode 1.

**Fig. 10:**
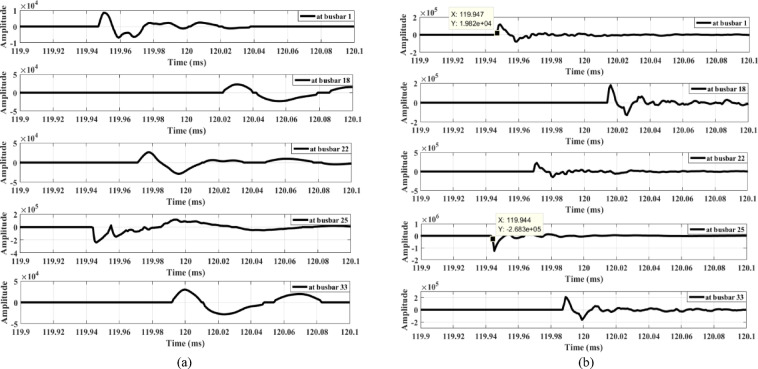
Extracted traveling waves under a fault F2: (**a**) zero mode, (**b**) aerial mode 1.

**Fig. 11:**
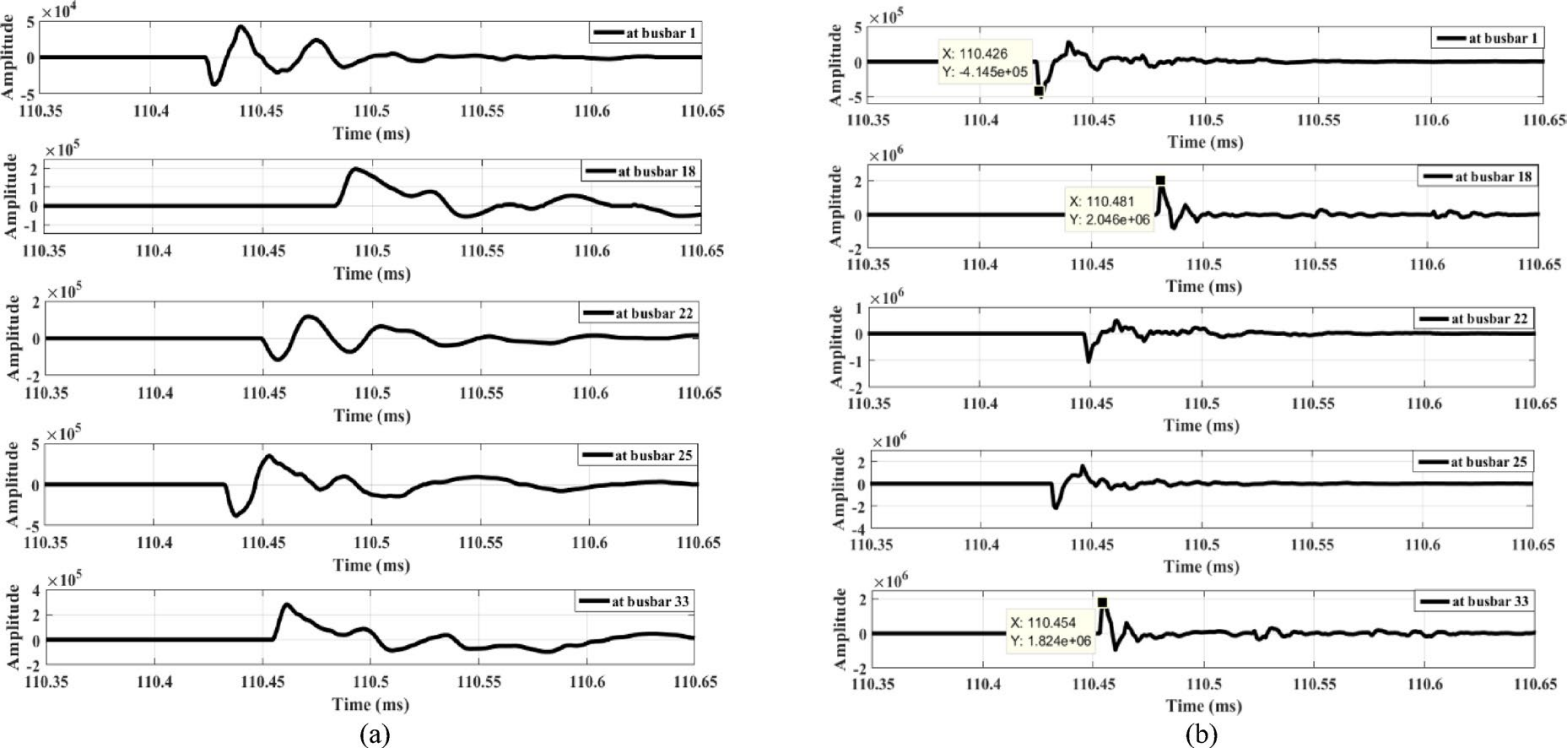
Extracted traveling waves under a fault F3: (**a**) zero mode, (**b**) aerial mode 1.

Figure 9 illustrates the signal derivative outputs for the zero mode and the aerial mode 1 of the measured voltage at busbar 1, along with the outputs from the lateral-end busbars (busbars 18, 22, 25, and 33) during fault F1. The data show that the polarity observed at busbar 33 is opposite to that of the other busbars, thereby confirming the detection of the fault and triggering the opening of the main feeder circuit breaker. Similarly, Fig. 10 illustrates the dependability of the proposed fault detection scheme during fault F2. The signal derivative outputs at busbar 25 exhibit a polarity distinct from that of the other busbars. This difference further substantiates the effectiveness of the detection method. In addition, the findings presented in Fig. 11 confirm the dependability of the detection scheme during fault F3, where the signal derivative outputs at busbars 18 and 33 show differing polarities compared to those of the other busbars.

### The fault management process

Table 3 summarizes the wave polarities at all considered measurement points under the three studied fault scenarios. Based on these polarities, the proposed scheme identifies the faulted lateral. Consequently, the agent at the faulted lateral end (the agent located downstream of the fault point) is identified. Then, the times of the first-arrival voltage waves at this agent and at the primary substation are then used to determine the faulted section and fault location. These times are summarized in Table 4 for the three studied fault scenarios. The details of these results are discussed in the following subsections.

**Table 3:** Polarities of the acquired traveling waves under different fault scenarios.

	Bus 1	Bus 18	Bus 22	Bus 25	Bus 33	Fault detection	Identified faulted lateral or region	
*F1*	-	-	-	-	+	√	From bus 6	To bus 33
*F2*	+	+	+	-	+	√	From bus 3	To bus 25
*F3*	-	+	-	-	+	√	From bus 3	To bus 6

**Table 4:** Calculation of the fault distance and determination of the faulted section.

Fault	Bus i-j	t_A_ (ms)	t_j_ (ms)	Calculated fault distance from busbar j	Determined fault section	Fault distance (% of determined fault section length)	Isolated faulted section(s)
*F1*	1–33	111.31	111.293	4.401	28–29	94%	28–29 and 29–30
*F2*	1–25	119.947	119.944	1.409	23–24	49.43%	23–24
*F3*	1–33	110.426	110.454	11.016	5–6	57.88%	5–6

#### Faulted lateral Estimation

As stated in the first row of Table 3, during fault F1, the polarity at busbar 33 is opposite to that of the other busbars. This indicates that the lateral containing busbar 33 is the faulted lateral, and the lateral end at busbar 33 is the only one downstream of the fault. Therefore, the faulted lateral starts from busbar 6 and ends at busbar 33.

Similarly, as presented in the second row of Table 3, during fault F2, a different polarity is observed only at busbar 25. Consequently, the lateral that includes busbar 25 is identified as the faulted lateral, with the lateral end at busbar 25 being the only one downstream of the fault. As a result, the faulted lateral extends from busbar 3 to busbar 25.

During fault F3, as presented in the third row of Table 3, a different situation arises because a different polarity is observed at both busbars 18 and 33, in contrast to all other measurement points (including busbar 1), which have the same polarity. This indicates that the fault is upstream of the intersection point of the laterals containing busbars 18 and 33, specifically at busbar 6, while being downstream of busbar 3. Therefore, the faulted region is located between busbar 3 to busbar 6.

#### Faulted section Estimation

After determining the faulted lateral, a two-terminal fault location scheme based on traveling waves is used to calculate the fault distance. The traveling-wave velocity used for the distance calculation is 294 km/ms^24^. Under the three considered fault cases, the times of the captured first-arrival waves at the primary substation (agent *A*) and at the lateral end (busbar *j*), *denoted as t*_*A*_ and *t*_*j*_, are recorded as shown in Table 4. For faults F1 and F2, the fault distance is calculated from the measurement point at the end of the faulted lateral (busbar *j*) using the formula presented in (2). For fault F3, the time reference can be taken from either busbar 18 or busbar 33, which serve as later ends downstream of the fault point. Therefore, a predefined dominant busbar is used as the time reference, with busbar 33 chosen as the dominant one in this case.

For faults F2 and F3, the calculated fault locations fall within Sections 23–24 and 5–6, respectively, at positions between 10% and 90% of these section lengths. In contrast, for fault F1, the calculated distance corresponds to about 90%–100% of the length of Section 28–29. Consequently, for faults F2 and F3, only the respective faulted section is isolated. However, for fault F1, both Sections 28–29 and 29–30 need to be isolated. The isolation is achieved by transmitting signals from the primary substation to the upstream and downstream switches adjacent to the target section(s), commanding them to open.

**Fig. 12:**
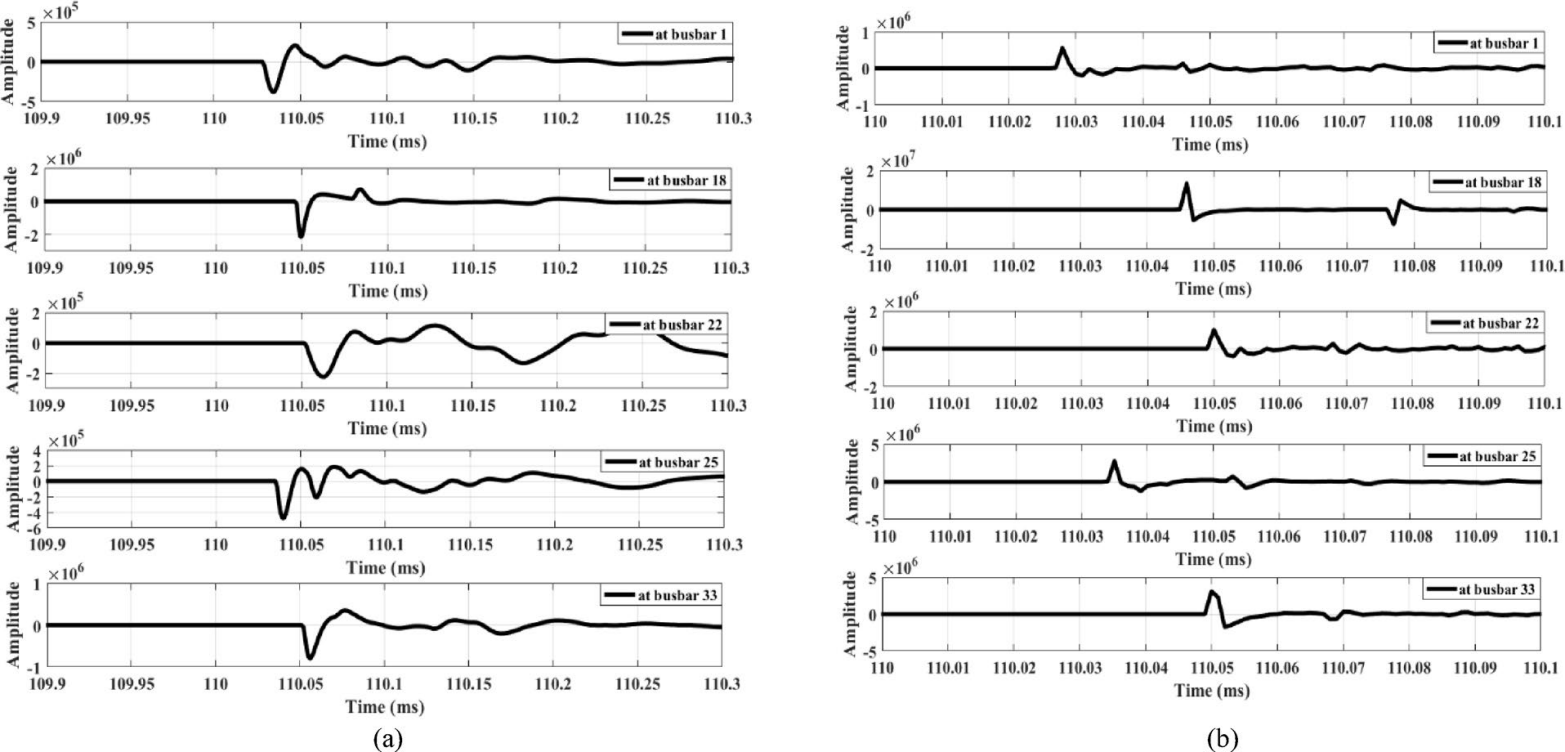
Extracted traveling waves under a high impedance earth fault: (**a**) zero mode, (**b**) aerial mode 1.

## Security assessment of the proposed series fault detection scheme

The security of the proposed scheme is evaluated under various normal and abnormal operating conditions that may occur in the distribution system. These conditions include shunt faults covered by other existing functions^22^, switching operations, synchronization errors, system unbalance, and external low-voltage side faults.

### Earth fault

An earth fault with 10,000 Ω fault resistance is applied between busbars 9 and 10. The extracted surges are shown in Fig. 12, where all waves exhibit high amplitudes. However, the waves from all measurement points have the same polarity. As a result, the proposed scheme remains secure under these shunt faults. This indicates that the proposed scheme is specifically designed for series faults and operates only in the presence of an open-conductor fault.

### Switching

The security of the proposed scheme is also evaluated under various switching conditions, including medium-voltage (MV) load disconnection with delta/star-earthed transformer configurations, MV load disconnection with star-earthed/star-earthed transformer configurations, low-voltage (LV) load disconnection with star-earthed/star-earthed transformer configurations, induction motor connection, capacitor bank connection at different practical levels, and the disconnection of a three-phase MV lateral at different pole opening times.

**Fig. 13:**
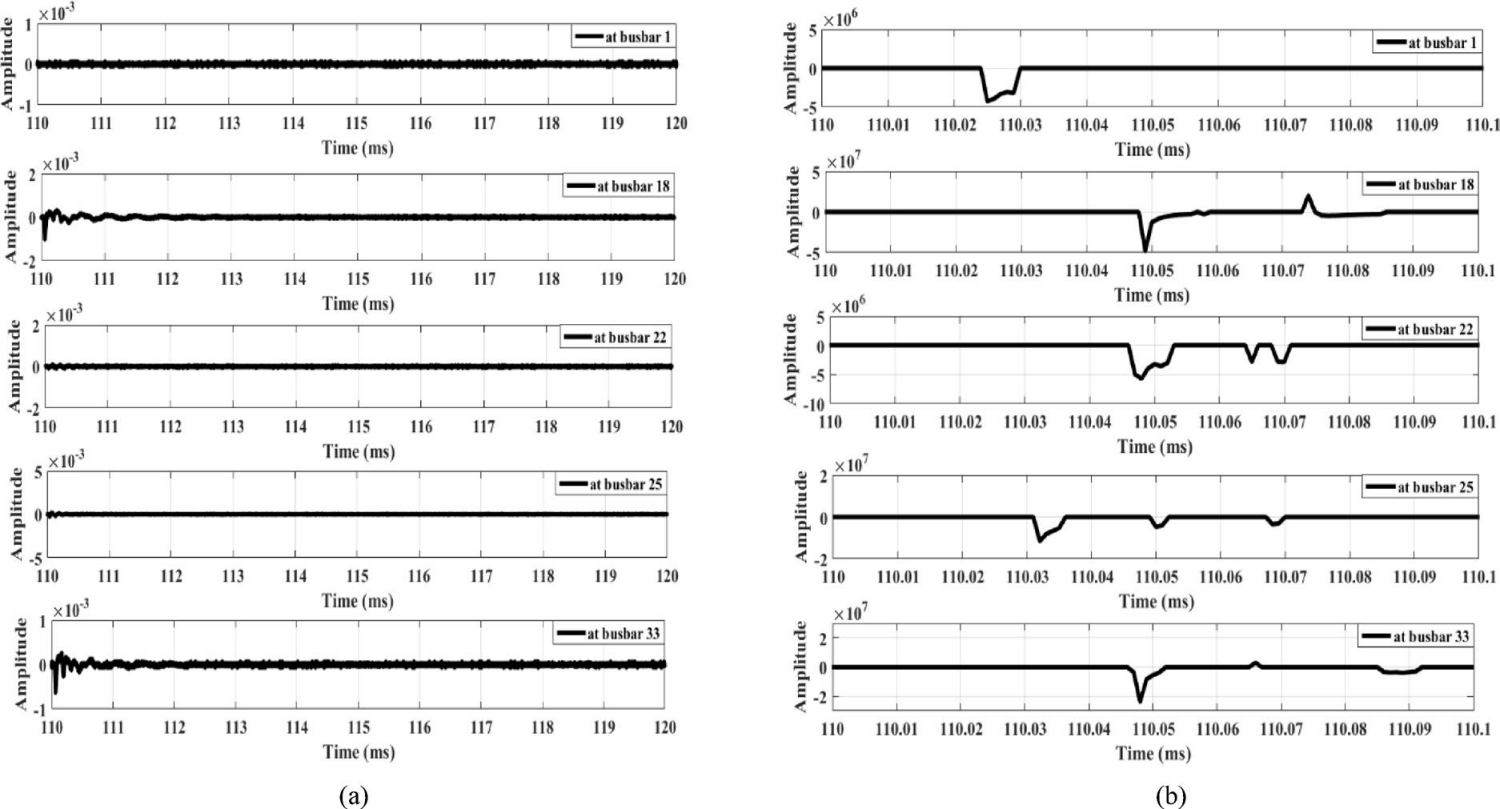
Extracted traveling waves during the disconnection of an MV load connected to a delta/star-earthed transformer: (**a**) zero mode, (**b**) aerial mode 1.

The first case examines the response of the proposed scheme when an MV load connected to busbar 9 through a delta/star-earthed transformer is disconnected, as shown in Fig. 13. Figure 13a shows the waves extracted from the zero-sequence mode, where the amplitudes are very small—almost zero. In contrast, Fig. 13b shows the waves extracted from the aerial mode, in which all waveforms exhibit the same polarity. Therefore, the proposed scheme remains secure during the disconnection of loads connected through delta/star-earthed transformers.

**Fig. 14:**
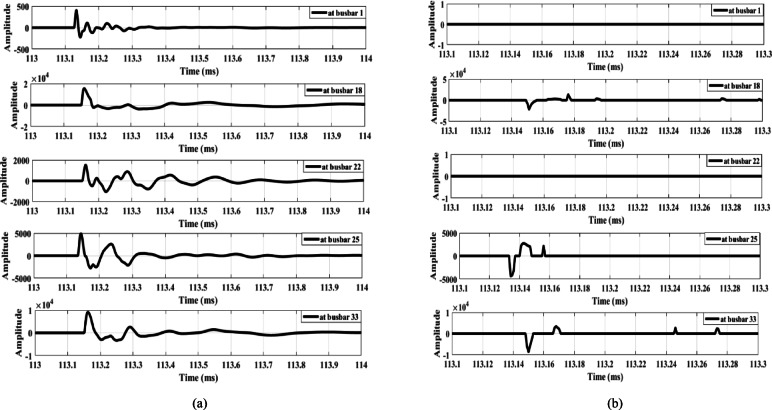
Extracted traveling waves during the disconnection of an MV load connected to a star-earthed/star-earthed transformer: (**a**) zero mode, (**b**) aerial mode 1.

**Fig. 15:**
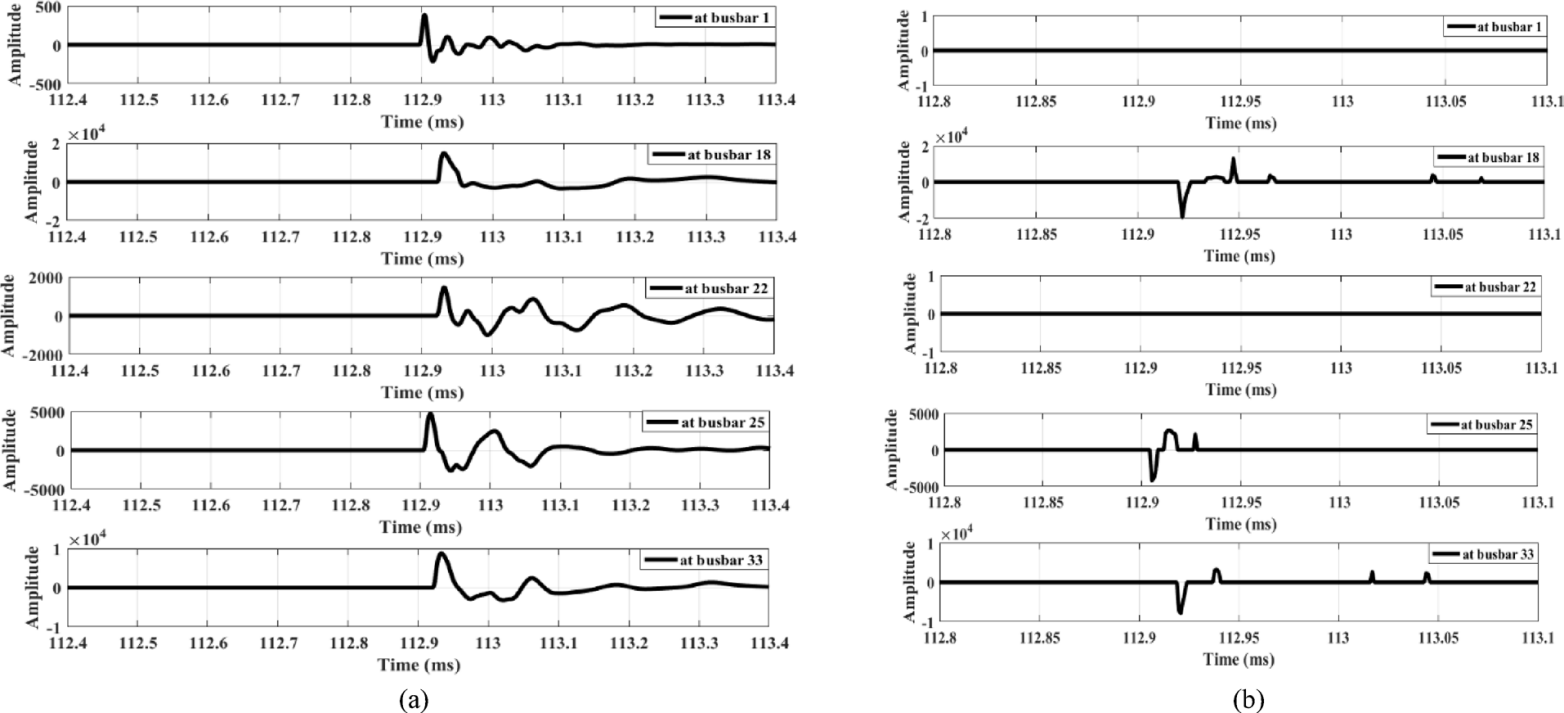
Extracted traveling waves during the disconnection of a LV load connected to a delta/star-earthed transformer: (**a**) zero mode, (**b**) aerial mode 1.

Similarly, when the same load is disconnected from the MV side through a star-earthed/star-earthed transformer configuration, the resulting waveforms are shown in Fig. 14. Additionally, Fig. 15 presents the waves generated when the load is disconnected from LV side. Both cases produce high-amplitude waves with identical polarities, confirming that the proposed scheme maintains its security under different load disconnection scenarios.

**Fig. 16:**
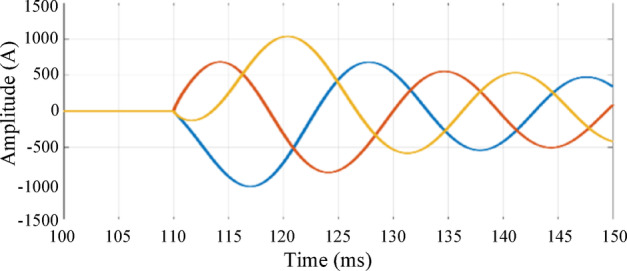
Three-phase current during the connection of an induction motor on the MV side.

**Fig. 17:**
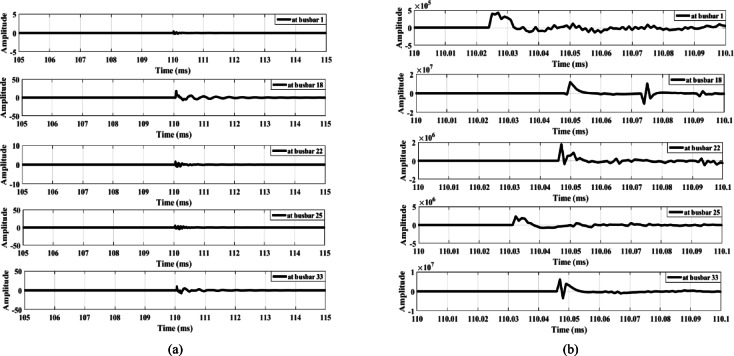
Extracted traveling waves during the connection of an induction motor on the MV side: (**a**) zero mode, (**b**) aerial mode 1.

The second condition involves connecting an induction motor to the MV side of the system at busbar 9 at 0.11 s. This scenario is examined to evaluate the effect of the motor’s starting transient period, as shown in Fig. 16, on the performance of the proposed technique. Figure 17a shows the waves extracted from the zero-sequence mode, whose amplitudes are too small to be detected. In addition, the waves extracted from aerial mode 1 exhibit the same polarity, as illustrated in Fig. 17.b. Therefore, the proposed scheme remains secure during the connection of induction motors.

**Fig. 18:**
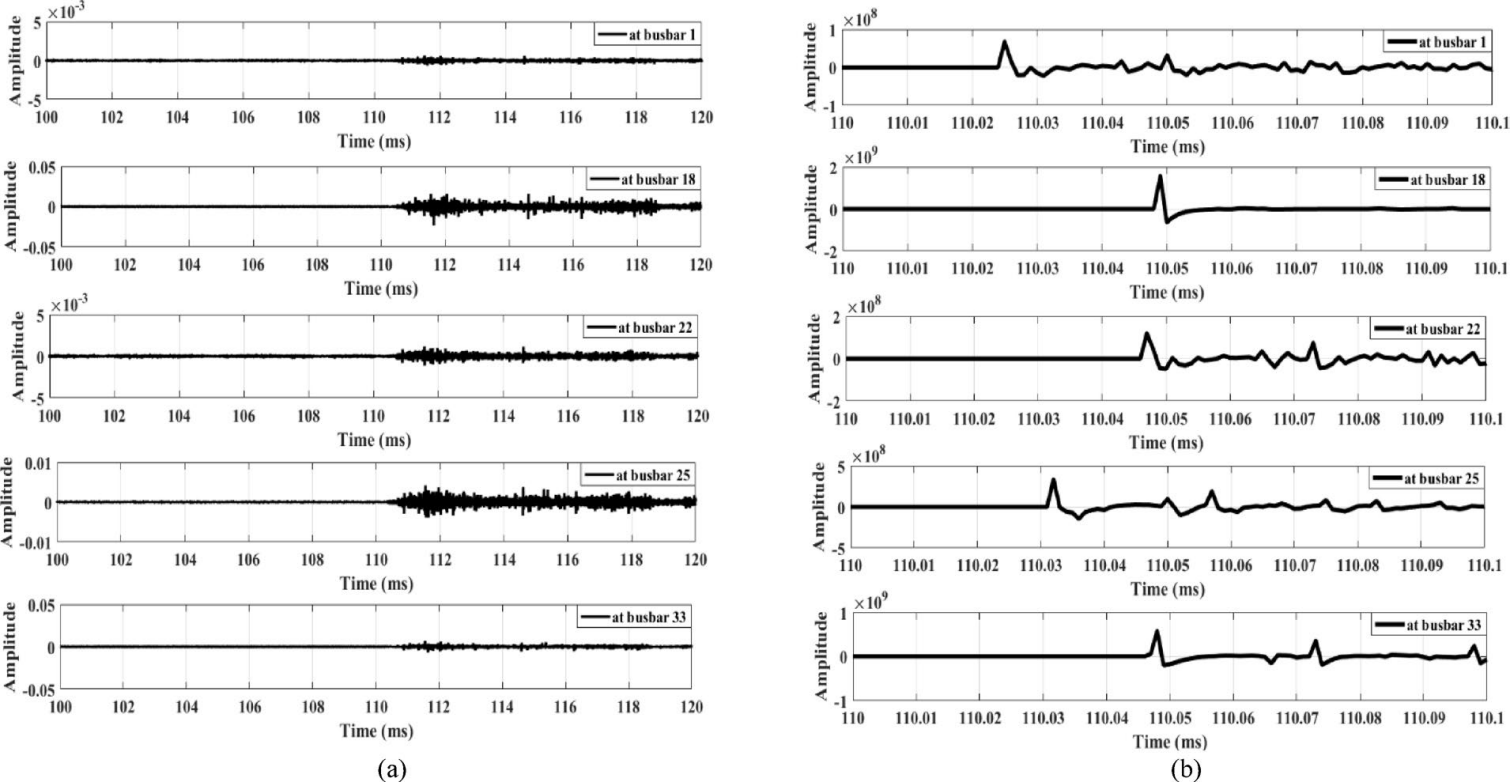
Extracted traveling waves during the connection of a 1.8 MVAR capacitor bank on the MV side: (**a**) Zero mode, (**b**) Aerial mode 1.

**Fig. 19:**
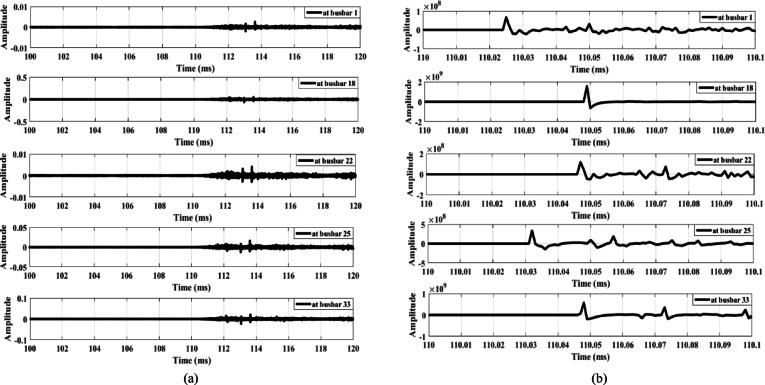
Extracted traveling waves during the connection of a 3.6 MVAR capacitor bank on the MV side: (**a**) Zero mode, (**b**) Aerial mode 1.

The third condition involves connecting a capacitor bank at busbar 9. Capacitor banks of different ratings—1.8 MVAR (Fig. 18) and 3.6 MVAR (Fig. 19)—are connected to the system. Figures 18a and 19a show that the wave amplitudes extracted from the zero-sequence mode, under connections of 1.8 MVAR and 3.6 MVAR capacitor banks, respectively, are low. Therefore, the proposed scheme is not triggered and does not operate. Furthermore, Figs. 18b and 19b, extracted from aerial mode 1 under the same connections, reveal that the waveforms exhibit the same polarity in both cases. Hence, the proposed scheme remains secure during the connection of capacitor banks of different ratings.

**Fig. 20:**
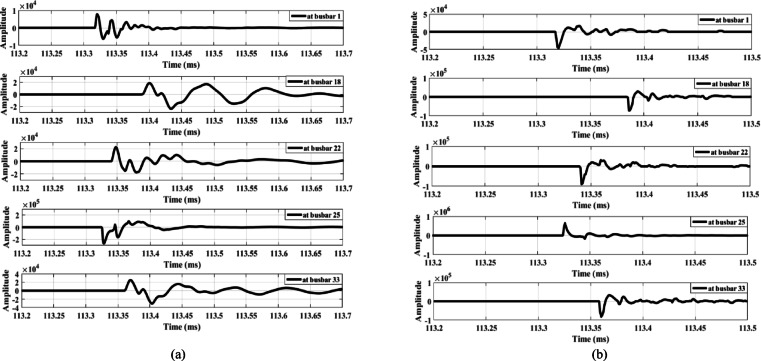
Extracted traveling waves during the disconnection of a three-phase MV lateral with different pole opening times: (**a**) zero mode, (**b**) aerial mode 1.

Furthermore, the performance of the proposed scheme is evaluated under the disconnection of a lateral between busbars 3 and 25, where the opening of the three poles does not occur simultaneously. A slight delay of a few microseconds between the openings of the three poles is simulated. During this interval, the network behaves similarly to a system subjected to an open-conductor fault. Consequently, the proposed scheme interprets this disconnection scenario as an open-conductor fault, recording differences in wave polarities (the polarity of the first-arrival wave at busbar 25 differs) with sufficient amplitudes, as shown in Fig. 20. To prevent maloperation, a coordination method is proposed between the load-switching case and the downstream agent at the end of the same lateral. In this method, a blocking signal is sent to the agent due to the switching operation, instructing the lateral end to temporarily stop sending its detected polarity status to the primary substation.

### Synchronization errors

The proposed scheme relies on capturing passive traveling waves generated by faults. These waves are collected from multiple endpoints, which makes the scheme potentially sensitive to synchronization misalignment between measurement points. However, the proposed fault detection method depends solely on the polarities of the waves and is therefore unaffected by synchronization errors. Similarly, identifying the faulted lateral, in the proposed fault management process, is based on comparing wave polarities from all ends, making it likewise immune to synchronization errors.

The only stage influenced by synchronization misalignment is the determination of the faulted section, which requires accurate time alignment between the measurements at the faulted lateral end and the primary substation. Tables 5 and 6 present the performance of the proposed faulted section identification under faults F1 and F2, respectively. Various synchronization errors were tested, including 1 µs, 2 µs, and 3 µs delays from one end. These errors were applied to both faults F1 and F2, where the distance to the fault—and thus the corresponding faulted section—was estimated.

As shown in Table 5, under fault F1 (busbar fault), the fault isolation can still be done in some cases even with a synchronization error of 1 µs. For faults occurring far from the end of the faulted section (fault F2), the proposed method correctly identifies the faulted section up to a synchronization error of 3 µs, as illustrated in Table 6.

Finally, the proposed scheme can detect and identify the faulted lateral independently of synchronization errors. Furthermore, any potential misidentification of the faulted section due to significant synchronization errors can be mitigated by verifying fault indicators at the identified secondary substations (as determined from the estimated distance) before isolating the corresponding section, thereby ensuring that the correct faulted section is ultimately isolated.

**Table 5:** Calculation of fault distance and determination of the faulted section under fault F1 and synchronization errors.

Error in t_A_	Calculated fault distance from busbar j	Determined fault section	Fault distance (% of determined fault section length)	Isolated faulted section(s)
1 µs	4.254	29–30	2.87%	28–29 and 29–30
2 µs	4.107	29–30	12%	29–30
3 µs	3.96	29–30	21.25%	29–30

Error in *t*_*j*_	Calculated fault distance from busbar *j*	Determined fault section	Fault distance (% of determined fault section length)	Isolated faulted section(s)
1 µs	4.548	28–29	85.4118%	28–29
2 µs	4.695	28–29	76.7647%	28–29
3 µs	4.842	28–29	68.1176%	28–29

**Table 6:** Calculation of fault distance and determination of the faulted section under fault F2 and synchronization errors.

Error in t_A_	Calculated fault distance from busbar j	Determined fault section	Fault distance (% of determined fault section length)	Isolated faulted section(s)
1 µs	1.2620	23–24	58.625%	23–24
2 µs	1.1150	23–24	67.8125%	23–24
3 µs	0.9680	23–24	77%	23–24

Error in *t*_*j*_	Calculated fault distance from busbar *j*	Determined fault section	Fault distance (% of determined fault section length)	Isolated faulted section(s)
1 µs	1.556	23–24	40.25%	23–24
2 µs	1.7030	23–24	31.0625	23–24
3 µs	1.85	23–24	21.875	23–24

### Unbalanced loading and low voltage side fault conditions

**Fig. 21:**
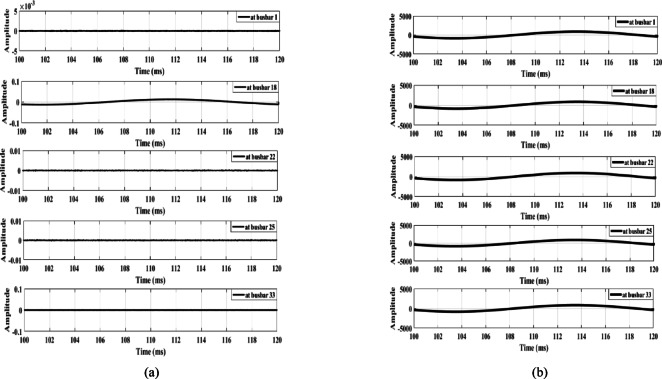
Extracted traveling waves under unbalanced load: (**a**) Zero mode, (**b**) Aerial mode 1.

**Fig. 22:**
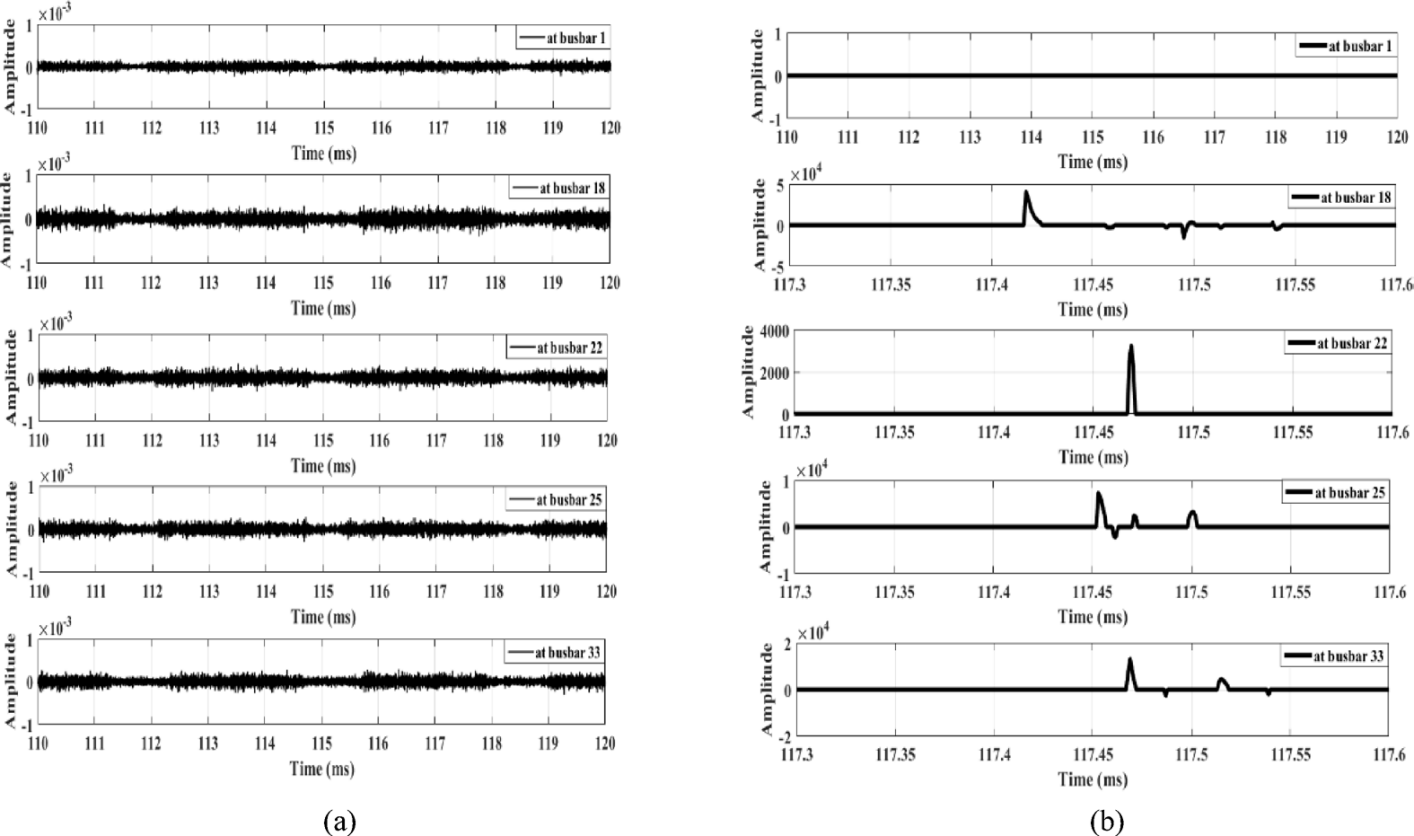
Extracted traveling waves for an open-conductor fault at the low voltage side of downstream busbar 15: (**a**) Zero mode, (**b**) Aerial mode 1.

The proposed scheme is tested under unbalanced load conditions, as shown in Fig. 21. The results indicate that the scheme remains unaffected even with an unbalance ratio of up to 40%. Additionally, the method is evaluated under an open-conductor fault on the low-voltage side downstream of busbar 15. The corresponding results, shown in Fig. 22, represent a fault occurring at the secondary terminals of the transformer connected to busbar 15. As illustrated, the wave amplitudes are very low, preventing any maloperation. These results demonstrate that the proposed method is secure under such conditions.

## Assessment of the proposed series fault detection method in the presence of distributed generation units

**Fig. 23:**
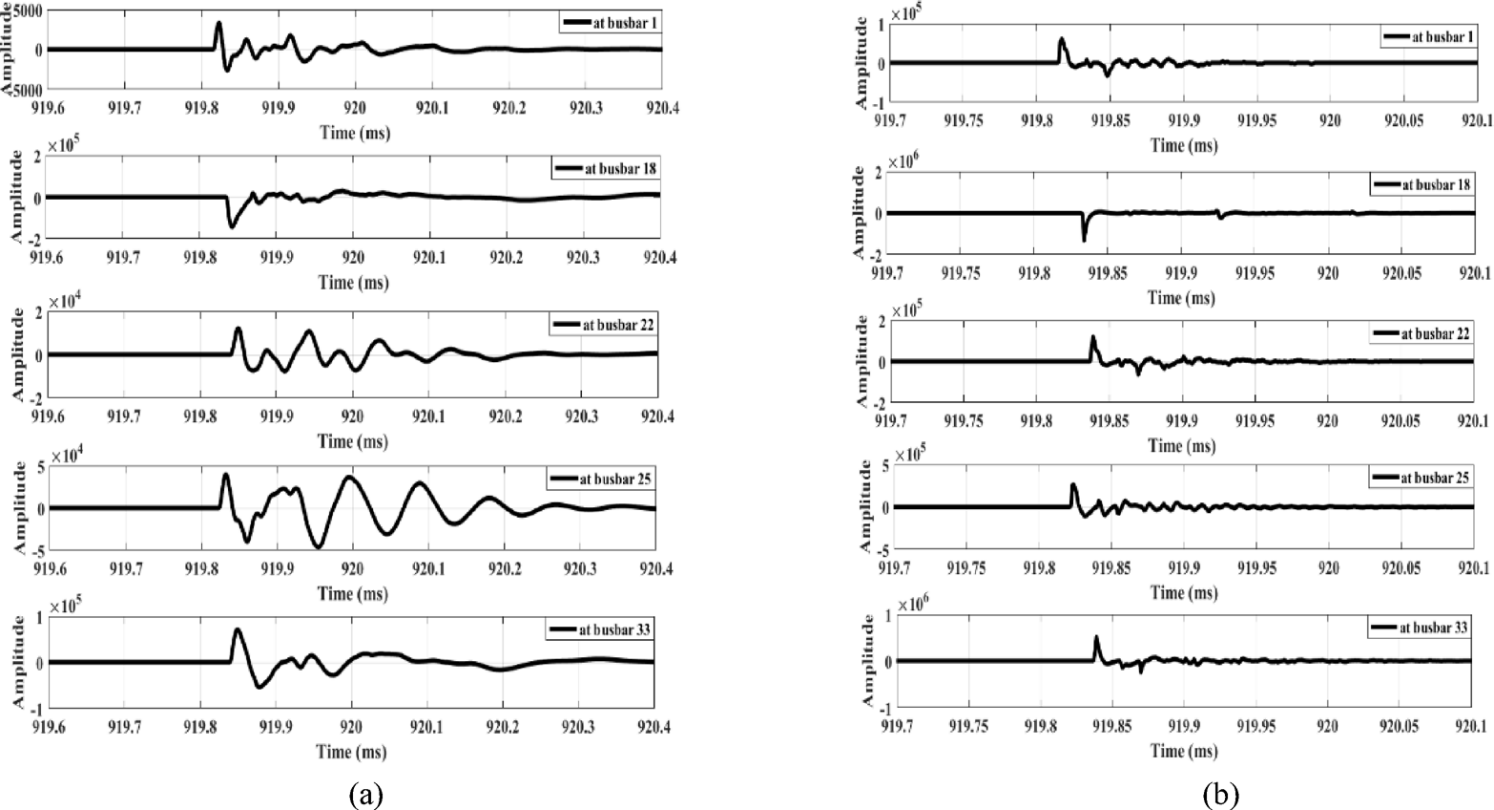
Extracted traveling waves for a fault between busbars 9 and 10 in the presence of DG at busbar 9: (**a**) zero mode, (**b**) aerial mode 1.

The proposed method is tested in the presence of a DG connected to the distribution system. Two test cases are considered, examining series faults upstream and downstream of the DG location. In the first case, an open-conductor fault is simulated between busbars 9 and 10 while a Type-2 wind farm generator, rated at one-third of the total system power, is connected at busbar 9—placing the fault downstream of the DG. Figure 23 shows the extracted waves produced by the fault. Figure 23 illustrates that a different polarity is observed only at busbar 18, unlike all other measurement points (including busbar 1), which exhibit the same polarity. This indicates the occurrence of an open-conductor fault and triggers the opening of the main feeder circuit breaker. Consequently, the lateral containing busbar 18 is identified as the faulted lateral, with the lateral end at busbar 18 being the lateral downstream of the fault. Therefore, the faulted lateral between busbars 6 and 18 is correctly identified, with wave polarities positive upstream and negative downstream.

**Fig. 24:**
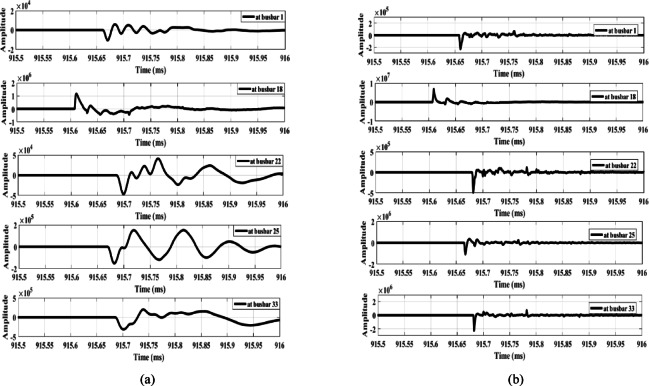
Extracted traveling waves for a fault between busbars 16 and 17 in the presence of DG at busbar 18: (**a**) zero mode, (**b**) aerial mode 1.

In the second case, the DG unit, at full rating, is connected at busbar 18, and an open-conductor fault is simulated upstream of the DG, between busbars 16 and 17. The extracted waves produced by this fault condition are shown in Fig. 24. Similarly, Fig. 24 shows a polarity change occurring only at busbar 18, where the wave polarity differs from all other measurement points, confirming the open-conductor fault. This condition triggers the opening of the main feeder circuit breaker and identifies the lateral containing busbar 18 as the faulted lateral. Therefore, the results shown in Figs. 23 and 24 confirm that the proposed series fault detection method is dependable in the presence of distributed generation units operating at full rating. Furthermore, its dependability is validated in Section V under zero DG output. Consequently, the proposed method is reliable across the full output range of DG units, from zero to full capacity, effectively accommodating uncertainties in renewable energy generation.

## Conclusions

This paper presents a novel series fault detection and management scheme designed specifically for modern distribution systems with high penetration of DG units. The proposed method utilizes the polarity of first-arriving traveling voltage waves at lateral ends to detect and locate series faults—including open-conductor and downed-conductor faults—that are often undetected by conventional protection systems. Unlike traditional methods, it requires only voltage measurements, does not rely on predefined threshold values, and is inherently robust under various system conditions.

The scheme is thoroughly tested using PSCAD simulations on the IEEE 33-bus system under various scenarios, including different fault types, load connection and disconnection events, and capacitor bank switching. The results confirm that such transient surges do not impact the security of the introduced scheme. Synchronization errors are also investigated, revealing that neither the detection stage nor the faulted lateral identification stage is affected. However, synchronization misalignments exceeding 1 µs may lead to inaccuracies only in the fault distance estimation. Therefore, incorporating a verification stage based on fault indicators at secondary substations mitigates errors in fault distance calculation and faulted section determination, thereby ensuring reliable isolation of the correct faulted section and enhancing overall system dependability.

Furthermore, the proposed scheme is evaluated in the presence of DG. It performs reliably across a wide range of DG contributions, from zero to full output, without requiring any adaptive tuning. The method successfully detects series faults regardless of the location of DG units, maintaining its reliability and accuracy where conventional techniques fail. Overall, the proposed scheme is highly sensitive to various types of series faults—both with and without DG—and secure under normal operating conditions. These results confirm its suitability for integration into active distribution networks, offering a scalable, dependable, and future-proof solution for fault detection, localization, and self-healing.

## Data Availability

All data generated or analyzed during this study are included in this published article.

## Abbreviations

DG: Distributed generation
MV: Medium voltage F2
LV: Low voltage F3
LE: Lateral end 1 PS
F1: First fault occurs at busbar 29
F2: Second fault occurs in Section 23–24
F3: Third fault (F3) occurs in Section 5–6
PS: Primary substation

## Variables

I₁: Positive-sequence current
I₂: Negative-sequence current
${v}_{\alpha\:}$: Aerial mode 1 voltage
${v}_{\beta\:}$: Aerial mode 2 voltage
$\:{v}_{0}$: Zero mode voltage
∆I: Current change
∆v: Voltage change
I_pre_: Pre-fault current
∆v_i_: Voltage change at upstream agents
∆i_busi_: Current change upstream of the fault
∆v_j_: Voltage change at downstream agents
∆i_DG_: Current change at the DG
d: Calculated distance
t_A_: Time of the first-arrival wave captured at PS
t_j_: Time of the first-arrival wave captured at lateral end j
